# Advancing Age Decreases Pressure-Sensitive Modulation of Calcium Signaling in the Endothelium of Intact and Pressurized Arteries

**DOI:** 10.1159/000454811

**Published:** 2017-01-19

**Authors:** Calum Wilson, Christopher D. Saunter, John M. Girkin, John G. McCarron

**Affiliations:** ^a^Strathclyde Institute of Pharmacy and Biomedical Sciences, University of Strathclyde, Glasgow, Durham, UK; ^b^Centre for Advanced Instrumentation, Biophysical Sciences Institute, Department of Physics, Durham University, Durham, UK

**Keywords:** Endothelium, Calcium signaling, Imaging, Aging

## Abstract

Aging is the summation of many subtle changes which result in altered cardiovascular function. Impaired endothelial function underlies several of these changes and precipitates plaque development in larger arteries. The endothelium transduces chemical and mechanical signals into changes in the cytoplasmic calcium concentration to control vascular function. However, studying endothelial calcium signaling in larger arteries in a physiological configuration is challenging because of the requirement to focus through the artery wall. Here, pressure- and agonist-sensitive endothelial calcium signaling was studied in pressurized carotid arteries from young (3-month-old) and aged (18-month-old) rats by imaging from within the artery using gradient index fluorescence microendoscopy. Endothelial sensitivity to acetylcholine increased with age. The number of cells exhibiting oscillatory calcium signals and the frequency of oscillations were unchanged with age. However, the latency of calcium responses was significantly increased with age. Acetylcholine-evoked endothelial calcium signals were suppressed by increased intraluminal pressure. However, pressure-dependent inhibition of calcium signaling was substantially reduced with age. While each of these changes will increase endothelial calcium signaling with increasing age, decreases in endothelial pressure sensitivity may manifest as a loss of functionality and responsiveness in aging.

## Introduction

The endothelium is a single-cell-thick layer that lines the inner surface of all blood vessels and is exposed to numerous activators, such as blood-borne signaling molecules, electrical signals, and mechanical forces. The endothelium responds to these activators to control vascular function by releasing vasoactive agents such as nitric oxide (NO) and prostaglandins and by initiating endothelium-dependent smooth muscle hyperpolarization. With advancing age, the ability of the endothelium to control vascular tone is attenuated in animal models and in humans [1,2,3,4]. This impairment is characterized by a decreased release of NO and relaxation of smooth muscle cells induced by endothelium-dependent vasoactive agonists such as acetylcholine (ACh) [5,6,7,8]. In addition to functional changes, aging is associated with significant structural changes in the arterial wall, including increased vessel stiffness, luminal diameter, and wall thickness [9,10,11]. The physiological and pathophysiological importance of structural changes is highlighted by the use of pulse wave velocity – an indicator of arterial stiffness [12] – as a predictor of cardiovascular mortality [13,14,15]. However, whilst several clinical end-points, such as pulse wave velocity, have been established to assess cardiovascular risk and guide treatment intervention, the underlying mechanisms responsible for functional changes in the arterial wall remain unclear.

A rise in endothelial cytoplasmic Ca^2+^ concentration ([Ca^2+^]_i_) is a major trigger for the agonist-induced production of NO [16,17,18]. Central, therefore, to an understanding of the endothelial control of the vasculature is an appreciation of Ca^2+^ signaling in native endothelial cells. Activation of several endothelial receptor systems initiates biphasic increases in intracellular Ca^2+^ that result from Ca^2+^ release from the endoplasmic reticulum (ER) and Ca^2+^ entry across the plasma membrane. For example, muscarinic receptor-mediated biphasic increases in [Ca^2+^]_i_ consist of an initial transient increase in intracellular Ca^2+^, due to inositol trisphosphate (IP_3_)-mediated release from the ER, followed by a sustained elevation maintained by Ca^2+^ influx from the extracellular space [19,20]. The age-dependent reduction in the production of NO may be causally related to altered endothelial Ca^2+^ signaling occurring via either influx or release. However, in cut open (en face) murine aorta, muscarinic (carbachol) IP_3_-mediated endothelial Ca^2+^ signaling was unaltered with increasing age [21]. In other studies, the sustained phase of the Ca^2+^ signal generated, by a maximal concentration of ACh, was increased in intact endothelial tubes isolated from the superior epigastric artery of aged mice [22]. This result suggests that Ca^2+^ influx activated by ACh (believed to occur via TRP channels [23,24]) was augmented with advanced age. However, somewhat unexpectedly, Ca^2+^ influx occurring via TRP channels (activated by H_2_O_2_) was reduced in aging animals as assessed by a ruthenium red block of the H_2_O_2_-induced Ca^2+^ rise [22].

In addition to chemical activators, endothelial signaling is regulated by mechanical forces such as pressure. Relatively little is known about how pressure affects endothelial Ca^2+^ signaling in intact arteries, and changes that occur with age are largely unknown. Recently, in intact pressurized arteries, we reported that the magnitude of agonist-evoked endothelial Ca^2+^ signals decreased as pressure was increased [25]. The pressure-dependent attenuation of endothelial Ca^2+^ signaling may underlie the reduced endothelium-dependent relaxation that occurs at increased intraluminal pressures in animal models [26,27,28] and in humans [29,30]. The pressure-sensitive decrease in Ca^2+^ signaling arose due to compression and flattening of endothelial cells which produced a restricted diffusive environment for Ca^2+^ release to proceed from the lumen of the store to the cytoplasm [25]. With advancing age, endothelial cells become thinner, and the luminal diameter of arteries increase [11,31,32]. As such, agonist-evoked endothelial Ca^2+^ signaling may be differently regulated in aged, compared to young, animals subject to similar mechanical loading.

In humans, significant cardiovascular changes occur into middle age, and so this is an important age group to study physiological changes. To investigate whether or not agonist-evoked and mechanical control of endothelial Ca^2+^ signaling was altered with age, we studied endothelial responses in intact and pressurized carotid arteries. Larger arteries, like the carotid artery, are sites where many of the most serious forms of cardiovascular diseases (e.g., atherosclerosis) develop, and these begin with endothelial dysfunction [33]. Furthermore, whilst often considered a simple conduit artery, the carotid artery contributes significantly to the control of cerebrovascular blood flow and cerebral vascular resistance [34,35]. In the present study, responses in aged (18-month-old) and young (3-month-old) rats were compared. Whilst relating rat ages to a human equivalent is not straightforward, 3-month-old rats are probably equivalent to young humans (∼10 years old) and 18-month-old rats equivalent to middle-aged humans (∼45 years old) [36].

Studying the effects of mechanical forces like pressure on endothelial function (and dysfunction) in larger arteries in a physiological configuration has been particularly challenging because of the difficulties in visualizing the endothelium through the thick artery wall. Assessment of endothelial function in large arteries has, as a result, been largely indirect [37]. In the present study we investigated endothelial [Ca^2+^]_i_ signaling responses to ACh using gradient index (GRIN) fluorescence endomicroscopy [25,38]. The results show that the sensitivity of endothelial cells to ACh is increased, while the pressure-dependent suppression of endothelial Ca^2+^ signaling is attenuated in the older rats (18 months). Thus, altered pressure-sensitive endothelial Ca^2+^ signaling may, at least in part, explain the reduction in endothelium-dependent responses (endothelial dysfunction) seen with age.

## Methods

### Ethical Approval

All animal care and experimental procedures were carried out with the approval of the University of Strathclyde Animal Welfare and Ethical Review Body and authorized under UK Home Ofﬁce regulations (Animals [Scientiﬁc Procedures] Act 1986, UK). Young (3-month-old) and aged (18-month-old) male Sprague-Dawley rats (250-350 g) were killed by overdose of pentobarbital sodium (intraperitoneal injection, ≥200 mg/kg; schedule 1 procedure; Animal [Scientific Procedures] Act 1986, UK). Subsequently, carotid arteries were quickly removed and placed in chilled physiological saline solution (PSS; pH 7.4) composed of (in mM): NaCl (145), KCl (4.7), MOPS (3-[N-morpholino]propane-sulfonic acid) (2.0), NaH_2_PO_4_ (1.2), glucose (5.0), EDTA (ethylenediaminetetraacetic acid) (0.02), MgCl_2_ (1.17), and CaCl_2_ (2.0).

### Microendoscopic Ca^*2+*^ Imaging and Analyses

**Fig. 1:**
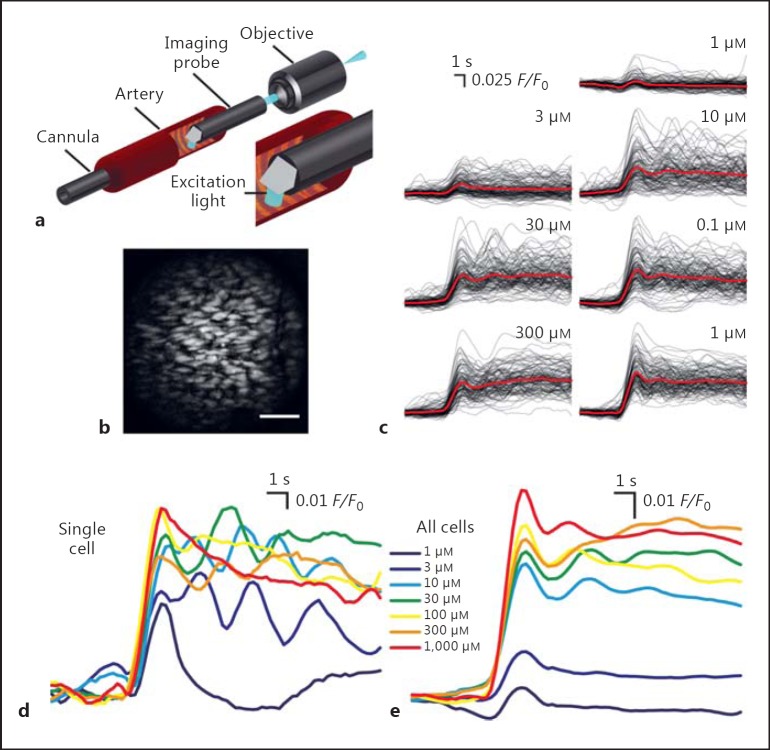
Population-wide concentration-dependent response in the endothelium of pressurized arteries from aged animals. **a** A simplified schematic diagram that illustrates GRIN microendoscopy of pressurized arteries. The cannula (left side) was used to pressurize the arteries. **b** An image of the endothelium obtained by probing the lumen of a pressurized (60 mm Hg) carotid artery from an aged (18-month-old) rat. Scale bar, 100 μm.**c** Representative baseline corrected and time-aligned (*F*/*F*₀) Ca^2+^ signals (black lines) and average (red lines; see online version for colors) of a population of endothelial cells, imaged across a single FOV, in response to various concentrations of ACh (60 mm Hg). **d** Individual traces of the Ca^2+^ levels in a single endothelial cell (from data shown in **c**) illustrating the evolution of the Ca^2+^ response of a single cell as the level of activation (ACh concentration) is increased. **e** Average endothelial Ca^2+^ activity (derived from traces shown in **c**), showing the increased Ca^2+^ responses to increasing ACh concentrations. The averaged response is the combined activity of the number of cells activated and the amplitude of the response in the field of cells.

Ca^2+^ signaling was monitored in the endothelium of pressurized arteries using GRIN microendoscopy (Fig. 1a, b), as previously described [25,38]. In brief, artery segments (∼20 mm long) were mounted onto cannula in a custom imaging bath, flushed with PSS for 10 min (150 µL/min) to remove blood, pressurized to 60 mm Hg, and then equilibrated at 37°C for 30 min. The endothelium was selectively loaded with a Ca^2+^ indicator by perfusing the lumen with PSS containing Oregon Green BAPTA-1/AM (20 µM; OGB-1/AM; No. O-6807; Invitrogen, Carlsbad, CA, USA) and Pluronic F127 (P-3000MP; Invitrogen). Once the Ca^2+^ indicator was introduced to the lumen, flow was stopped, and the endothelium allowed to load for 30 min. The final (working) concentrations of Pluronic F127 and DMSO were 0.04 and 0.96%, respectively. Following loading, excess dye was then flushed from the lumen (10 min; 150 µL/min), the distal cannula was removed, and the artery was mounted onto a side-viewing GRIN microendoscopic-imaging probe and repressurized. Throughout the loading procedure, the artery was continuously superfused with PSS that was warmed to 37°C before entering the bath.

Following equilibration, arteries were stimulated by direct application of ACh, delivered to the bath by a handheld pipette. A 30-s baseline period was recorded before each application of ACh and Ca^2+^ responses were recorded for 60 s after addition of ACh to the bath solution. Noncumulative concentration response experiments were carried out in arteries pressurized to 60 mm Hg; 60 mm Hg was selected because it is an exceptionally widely used pressure in myograph studies permitting comparison of the present results to the findings of others. During all imaging experiments, superfusion was halted to avoid movement artifacts, and the reported concentrations of ACh are final bath concentrations. Note that the ACh concentration when applied to the outside of the artery is 1,000 fold higher than required when ACh has free access to the endothelium (i.e., in an en face preparation [38]). Following each image acquisition, superfusion was recommenced (5 ml × min^−1^) to wash ACh from the bath, and arteries were allowed to reequilibrate for 20 min before the next stimulation. Importantly, in these experiments, the response of each individual endothelial cell was matched across each ACh concentration. The response of individual cells was normalized to the maximal averaged response. Endothelial Ca^2+^ signaling was also assessed at various intraluminal pressures. In pressure experiments, arteries were first stimulated with 100 µM ACh at 60 mm Hg, as this concentration was found to activate the majority of cells in the field of view (FOV). Following washout of ACh, intraluminal pressure was increased, from 60 to 110 mm Hg and subsequently from 110 to 160 mm Hg. Arteries were allowed to equilibrate at each experimental pressure for 20 min before stimulation. In the pressure change experiments, matching of individual cells was not possible because of cell movement that occurred as artery diameter changed. The response of individual cells was normalized to the maximal averaged response at 60 mm Hg. In all experiments, images were acquired at 5 Hz using µManager software [39].

### Data Analysis and Statistics

A single carotid artery was studied from each animal. Ca^2+^ signals from individual endothelial cells were extracted using a semi-automated image processing procedure, as previously described [25,38]. Fluorescence signals were expressed as baseline-corrected values (*F*/*F*₀), calculated by dividing the raw signals by the average value of a 50-frame (10-s) preceding ACh-evoked Ca^2+^ activity. For visual clarity, *F*/*F*₀ signals were aligned with respect to their peak rate of change using a custom analysis script written in Python [25,38]. For each cellular signal, our custom Python script calculated derivate Ca^2+^ signals (*d*(*F/F*₀)/*dt*) and extracted the magnitude of the peak rate of change and the time at which it occurred. A cell was considered “active,” i.e., exhibited a Ca^2+^ transient, if the magnitude of the peak rate of change exceeded 3 times the noise level in the same cell. The noise level was defined as the standard deviation of that cell's rate-of-change signal during the baseline period. If a cell was considered active, peak *F/F*₀ values were measured automatically from the maximum *F/F*₀ value that occurred within 5 s (25 frames) following the time at which the peak in the derivate signal occurred – a time long enough for the peak *F/F*₀ value to have occurred and the transient usually to have started declining. The peak *F/F*₀ values (expressed as change in baseline-corrected fluorescence intensity; Δ*F*/*F*₀) thus represent the maximal *F/F*₀ value of each cell's response immediately following the initiation of Ca^2+^ activity, i.e., the magnitude of the initial ACh-induced peak. Summary Δ*F*/*F*₀ values are expressed as means ± SEM of *N* cells from *n* animals. Curves were fitted to normalized concentration response data using Graphpad Prism 6.0 (GraphPad Software, USA). The maxima and minima of the curves were constrained to unity and zero, respectively. Calculated curve-fit parameters (half maximal effective concentration; EC_50_) are presented with 95% confidence intervals and were compared statistically using the extra sum-of-square F test.

To objectively analyze Ca^2+^ oscillations, peaks were identified from derivate Ca^2+^ traces (*d*(*F*/*F*₀)/*dt*), using a “zero-crossing detection” algorithm written in Python. The zero-crossing detector identified peaks that rose more than 3-fold the standard deviation of baseline (the first 50 frames of the recording) noise and provided a list of times of “zero crossing” which were organized into sequential pairs. The sign of the critical point of the derivate signal between each pair was used to determine whether the preceding “zero crossing” corresponded to a peak or nadir in the *F/F*₀ signal. The zero-crossing detection algorithm thus provided the time of each peak in each Ca^2+^ which was used to automatically extract conventional measurements (e.g., amplitude) from the corresponding *F/F*₀ data. Oscillatory cells were defined as those exhibiting two or more peaks (initial ACh-induced peak plus at least one additional oscillation peak). The fractions of oscillatory cells in young and aged animals were compared statistically using 1-way ANOVA. To analyze oscillation frequencies, the number of peaks occurring within a 60-s period after ACh-induced activation was calculated for each oscillatory signal and compared using a hierarchical (nested) 1-way ANOVA. Curves were fitted to normalized temporal distributions (jitter profiles) using Origin 9.1. The center of curves and peaks were constrained to zero and unity, respectively. Calculated curve-fit parameters (full width at half maximum, FWHM) were compared statistically using the extra sum-of-square *F* test. Pressure data (peak Δ*F*/*F*₀) were compared statistically using 2-way nested ANOVA (with Tukey's post hoc test as appropriate) in Minitab 17 (Minitab Inc., USA). Biologic replicate (animal) was treated as a random effect. A value of *p* < 0.05 was considered statistically significant in all tests.

## Results

### Effect of Age on Concentration-Dependent ACh-Evoked Endothelial Ca^*2+*^ Signaling

**Fig. 2:**
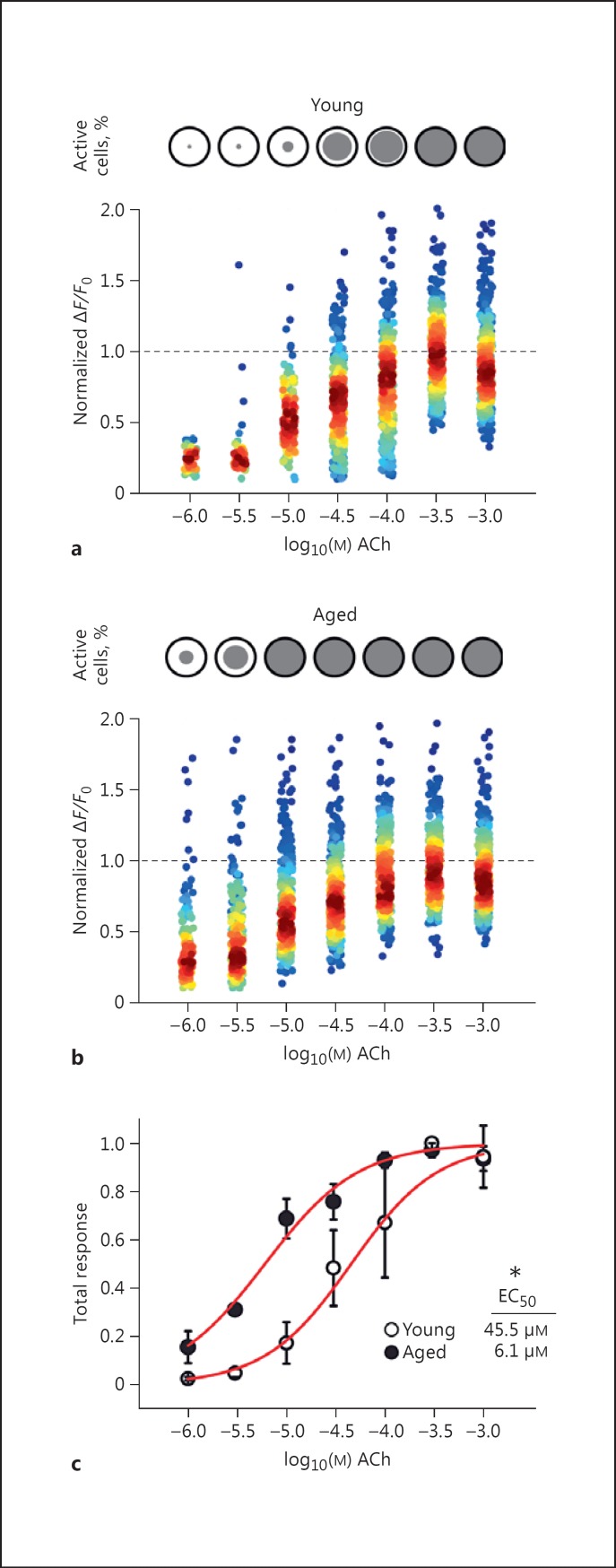
Aging enhances endothelial agonist sensitivity. **a** Summary data illustrating the percentage of endothelial cells (top) and normalized peak Δ*F*/*F*₀ values for individual cells (bottom) activated by each concentration of ACh. The percentage of cells activated by each concentration of ACh cells (gray circle) is shown in comparison to the maximal number of cells (black outlined circle). Normalized peak Δ*F*/*F*₀ values of active cells are pseudocolored according to the density of plotted scatter points (per ACh concentration; low-high, blue, green, yellow, red; see online version for colors). **b** Percentage of active endothelial cells (top) and corresponding normalized peak Δ*F*/*F*₀ values for individual cells (bottom panel) from aged animals, illustrated as in **a**. **c** Summary data illustrating the total endothelial response (peak Δ*F*/*F*₀ of active cells averaged across the total population) for young and old. Summary data are means ± SEM; 445 and 379 cells from 3 young and 3 old animals, respectively. * *p* < 0.05. All data were obtained at a pressure of 60 mm Hg.

Endothelial cells of intact and pressurized rat carotid arteries from young animals exhibit heterogeneous concentration-dependent rises in [Ca^2+^]_i_ in response to ACh (Fig. 2a) [38]. The Ca^2+^ response across the endothelium of arteries from aged animals was also heterogeneous (Fig. 1). Increasing ACh concentration (1 μM to 1 mM) resulted in both a graded increase in the number of cells activated and the amplitude of response in each cell (Fig. 1c, d, 2b). Note that the ACh concentration when applied to the outside of the artery is ∼1,000-fold higher than required when ACh has free access to the endothelium (i.e., in an en face preparation [38]). The temporal characteristics of the Ca^2+^ signals also evolved as the ACh concentration increased. Transient Ca^2+^ increases occurred at lower ACh concentrations (e.g., 1 µM), and sustained increases with repetitive oscillations occurred at higher ACh concentrations (e.g., above 3 µM; Fig. 1d). While the behavior of individual cells was complex, the aggregate Ca^2+^ response of the endothelial cell population was a smoothly graded [Ca^2+^]_i_ increase with ACh concentration (Fig. 1e, 2c). To illustrate the *total* endothelial response, peak Δ*F*/*F*₀ values were averaged across the endothelial cell population (Fig. 1e; analogous to traditional photometry measures). Inspection of the data from aged animals revealed that endothelial cells were more sensitive to ACh, and a higher percentage of cells responded at lower ACh concentrations than young animals (Fig. 2a, b: top, circles). To enable comparison of the overall sensitivity of the endothelium in young and aged animals, data from individual experiments were normalized to maximal response and averaged across experiments. The endothelium of aged (EC_50_ = 6.1 μM; 95% confidence interval 4.4-8.3 μM*; n* = 3) rats was significantly more sensitive than that of young (EC_50_ = 45.5 μM; 95% confidence interval, 27.0-76.6 μM; *n* = 3) rats (*p* < 0.05).

### Effect of Age on Temporal Properties of ACh-Evoked Endothelial Ca^*2+*^ Signaling

**Fig. 3:**
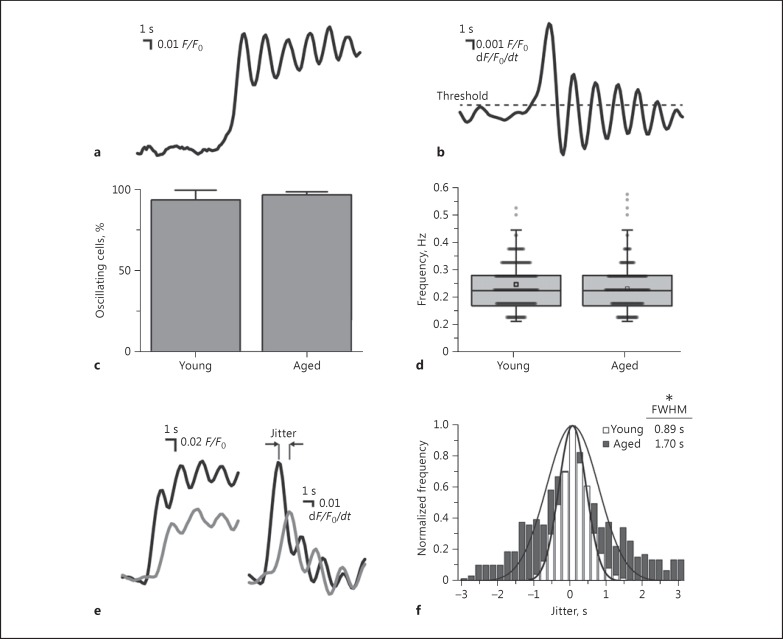
Age alters the temporal characteristics of endothelial Ca^2+^ signals. **a** Representative Ca^2+^ (*F*/*F*₀) signal from a single endothelial cell of a pressurized artery (60 mm Hg) stimulated with 100 μM ACh. **b** Derivate of the data shown in **a**. Oscillation frequency was determined from the peaks in the derivate signal (*d*(*F*/*F*₀)/*dt*; see Methods). **c** Summary data (means ± SEM) indicating the percentage of endothelial cells that exhibit oscillatory Ca^2+^ responses to 100 μM externally applied ACh (artery pressure 60 mm Hg; 648 and 578 cells from 5 young and 5 old animals, respectively). **d** Summary boxplots, with individual data points overlaid, illustrating the distribution of frequencies of ACh-evoked (100 μM externally applied) endothelial Ca^2+^ signals in young (606 cells from 5 animals) and old (559 cells from 5 animals) rats. The box depicts the interquartile range divided by the median, with the mean indicated by a square, and whiskers extend to a maximum of 1.5 times the interquartile range beyond the box. **e** Two representative unaligned Ca^2+^ (*F*/*F*₀) signals (left) and corresponding derivate signals (right) illustrating temporal “jitter” – the time between peak activation of individual cells. **f** Summary of frequency distribution illustrating the temporal jitter in young (948 cells from 8 animals) and aged (616 cells from 6 animals) endothelium. * *p* < 0.05.

As the concentration of ACh was increased, the endothelial Ca^2+^ response evolved from a transient Ca^2+^ increase that quickly returned to baseline, to a sustained increase with multiple oscillations (Fig. 1d). To determine if the increased sensitivity to ACh with age arose from altered kinetic properties of endothelial Ca^2+^ signals, which may indicate altered muscarinic receptor activation or altered communication between cells, we assessed the temporal properties of the Ca^2+^ responses in the endothelium of young and aged rats in response to a concentration of ACh (100 µΜ) sufficient to activate the majority of cells across the FOV. Temporal metrics were calculated from discrete derivate Ca^2+^ signals (Fig. 3a, b) using a zero-crossing detection algorithm (see Methods). Neither the percentage of oscillating cells (Fig. 3c; 93.5 ± 0.06%, 648 cells from 5 young animals; 96.7 ± 0.02%, 578 cells from 5 aged animals; *p* = 0.63) nor the frequency of oscillations (Fig. 3d; 0.24 ± 0.03 Hz; 606 cells from 5 young animals; 0.23 ± 0.02 Hz, 559 cells from 5 aged animals; *p* = 0.73) differed between young and aged rats. However, the temporal spread of the Ca^2+^ responses (latency; Fig. 3e, f) was significantly larger in aged compared to young animals (FWHM: 0.89 ± 0.03 s, young 60 mm Hg; 1.70 ± 0.11 s, aged 60 mm Hg). This increased temporal spread of Ca^2+^ responses in the endothelium of young animals may reflect altered diffusion of ACh through the artery wall or differences in the mechanisms initiating a Ca^2+^ rise in each cell.

### Effect of Age on the Pressure Dependence of ACh-Evoked Endothelial Ca^*2+*^ Signaling

**Fig. 4:**
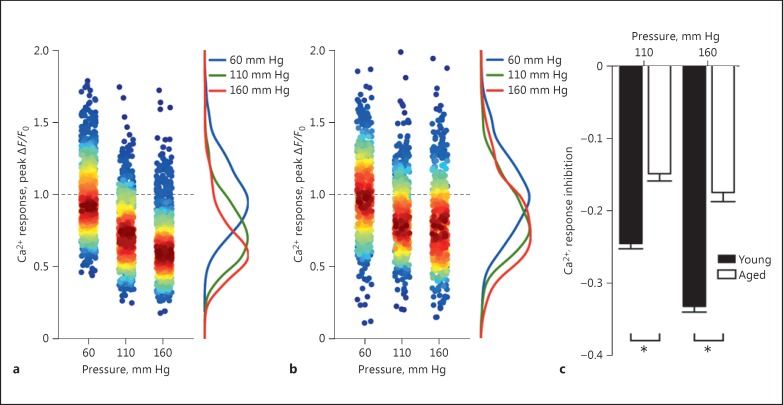
Age extenuates the effects of pressure on endothelial Ca^2+^ signaling. Summary data illustrating the pressure dependence of the Ca^2+^ response (peak Δ*F*/*F*₀) of endothelial cells activated by ACh in young (**a**; 948 cells from 8 animals) and aged rats (**b**; 616 cells from 6 animals). Cellular responses have been normalized to the average response occurring at 60 mm Hg. The responses are colored according to the density of plotted data points, and distributions obtained by kernel density estimation are shown for each pressure (see online version for colors). **c** Summary data illustrating inhibition of endothelial Ca^2+^ signaling in young and aged rats. Ca^2+^ response inhibition (means ± SEM) are defined as the difference between the response at the indicated pressure and control values. * *p* < 0.05.

ACh-evoked endothelial Ca^2+^ signaling is suppressed by increases in intraluminal pressure [25]. To examine the effects of aging on the pressure-induced suppression, the endothelial Ca^2+^ response to ACh (100 μM) was examined in individual arteries from aged animals, subject to stepwise pressure changes (60, 110, and 160 mm Hg) and compared to the pressure response of young animals. Like the response in young animals (Fig. 4a), endothelial Ca^2+^ signaling in arteries from aged animals was significantly decreased with increasing transmural pressure (Fig. 4b). However, the inhibition of ACh-evoked endothelial Ca^2+^ signaling at increased pressures was significantly reduced in the endothelium of aged versus young rats (Fig. 4c). Thus, the endothelium of aged animals is less sensitive to pressure changes than the endothelium of young animals.

## Discussion

Changes in arterial structure, associated with aging, increase tissue stress and degrade vascular function. Among the cellular components of the vascular wall, endothelial cells may be the most predisposed to age-related structural alterations. Endothelial Ca^2+^ signaling critically regulates blood vessel function across the entirety of the vasculature. Increases in endothelial [Ca^2+^]_i_ may trigger the release of vasodilators such as NO and prostaglandin or the spread of hyperpolarization to smooth muscle cells. Intrinsic hemodynamic forces, such as pressure, critically regulate the properties of Ca^2+^ signals in the endothelium and may be merged into the response evoked by agonists [25]. However, it is unclear if age-induced alterations in structural features of the artery impact directly upon endothelial Ca^2+^ signaling evoked by mechanical forces. Here, the regulation of endothelial Ca^2+^ signaling by pressure and agonists was studied in the endothelium of intact arteries of aged (18-month-old) rats and compared to that in young (3-month-old) rats. The results show that while the endothelium was more sensitive to ACh, pressure-dependent modulation of agonist signaling is attenuated with age. These alterations both contribute, paradoxically, to the increase in endothelial Ca^2+^ signaling with age.

### Mechanical Forces on the Vessel Wall

The two major mechanical stimuli which govern endothelium-dependent responses are shear stress and pressure [18,27,40,41,42,43,44], and responses to these stimuli change into middle age. The endothelial response to shear stress is well characterized and associated with *activation* of the endothelium. The response of the endothelium to pressure is less well understood, but differs significantly from that of shear stress. Indeed, a *decrease* in activity occurs as pressure is increased [26,45]. The decrease in endothelial activity may cause long-lasting inhibition of endothelium-dependent dilation in human volunteers [29,30] and reduced endothelium-dependent relaxation in experimental animals [26,27,28]. The mechanism underlying pressure-induced suppression of endothelial Ca^2+^ activity may be a reduced IP_3_-evoked Ca^2+^ release [25] or, alternatively, a decrease in TRPV4 activity [46]. Reduced IP_3_-evoked Ca^2+^ release with increasing pressure may occur from alterations in the diffusive environment governing Ca^2+^ release through IP_3_ receptors (IP_3_Rs). As pressure is increased, endothelial cells are flattened, and the distance between the ER and the opposing plasma membrane reduces and restrict Ca^2+^ diffusion away from IP_3_R [25]. As a result, [Ca^2+^]_i_ is increased (locally) at IP_3_R clusters following the release of the ion because of the restricted diffusional space. The increased local [Ca^2+^]_i_ at IP_3_R reduces the entropic force driving Ca^2+^ release and so decreases total Ca^2+^ liberated from the ER [25].

In middle age, structural changes occur across the artery wall. Endothelial cells become thinner, arterial luminal diameter increases, and arteries become less compliant [11,31,32]. These structural changes alter both the geometry of endothelial cells and the extent to which shape changes may occur with altered pressure. It is tempting to speculate that restrictions on the magnitude of endothelial shape changes in aging animals as a consequence of arterial structural changes may limit alterations in the Ca^2+^ diffusive environment and thus also the pressure-induced reduction in IP_3_-evoked Ca^2+^ signals.

Most studies on the changes in endothelial function with age have examined endothelial function indirectly using changes in contractile responses in intact tissue. The reported effects of age on endothelial function are varied when contractile responses are measured. Vascular relaxation responses are impaired in some studies in mice [47,48] but unchanged in others [49]. In rat aorta, the relaxation to muscarinic activation is reported to be decreased in rats aged 20 months [50], 22 months [51], and 24 months [3], but unaltered in rats aged 11-13 months [52]. Relaxation was also reported to be unaltered in aging (12 months) rabbit aorta [53]. Several mechanisms may contribute to endothelium-dependent alterations in contractile function regardless of differences in the age and severity of age-related pathology of the animals under investigation. Detectable differences within and across studies may also be due to the sensitivity and specificity of the research strategy used (e.g., vessels with intravascular pressure vs. no pressure and various types of microscopy used – see below).

The effects of age on endothelial Ca^2+^ signaling has been directly assessed and is reported to be altered in some, but not all, studies. Local (unstimulated) endothelial Ca^2+^ signaling is attenuated in mouse mesenteric arteries with advanced age (∼3-6 vs. 24 months corresponding to ∼25-35 vs. 70 years in humans) [54]. In mouse superior epigastric arteries, endothelial Ca^2+^ responses evoked by a single concentration of ACh were found to be increased with age (∼3-6 vs. 24 months; i.e., ∼25-35 vs. 70 years in humans) [22]. However, global Ca^2+^ responses evoked by muscarinic activation are unaltered by increasing age in mouse aorta (10 vs. 24 weeks; i.e., ∼25 vs. ∼35 years in humans) [21]. The main focus of the latter study was atherosclerosis development, and hence younger animals were used. It is possible that changes in the ACh-evoked Ca^2+^ response do not occur until age is more advanced [21]. In the present study in rat carotid arteries, endothelial cell sensitivity to Ach is increased in aged animals (3 vs. 18 months; i.e., ∼10 vs. 45 years in humans).

Thus, in the endothelium, there appears to be a decrease in unstimulated local responses [54] and an increase in ACh-evoked global Ca^2+^ responses [22]. Local and global Ca^2+^ signals may target different cellular mechanisms. For example, local Ca^2+^ signals in endothelial projections may provide negative feedback to smooth muscles via the activation of Ca^2+^-activated K^+^ channels [55]. Global signals may additionally generate NO via eNOS activation [56]. The difference in local and global signals that arise in aging animals raise the possibility that a diverse set of functional changes occur with increasing age from altered endothelial Ca^2+^ signals, e.g., impaired myoendothelial communication and increased NO production.

The mechanisms underlying endothelial sensitivity to ACh are complex. ACh-evoked, concentration-dependent Ca^2+^ responses arise from the heterogeneous responses of individual endothelial cells integrated across the entire endothelial cell population. In the present study of pressurized large arteries, only small subpopulations of endothelial cells are activated at low concentrations of ACh, (e.g., 1 µM in the bath, which is equivalent to 1 nM at the endothelium [38]). As the concentration of ACh is increased, recruitment of additional endothelial cells occurs. Each cell responds with a graded increase in Ca^2+^. However, each cell has a limited sensitivity to ACh. Population-wide heterogeneity regarding the sensitivity to the agonist extends the dynamic range over which the entire endothelium responds to ACh, from a single order of magnitude (in individual cells) to multiple orders of magnitude (across the total endothelial complement). The averaged activity generates a smoothly graded response to ACh. In the endothelium of aged rats, the sensitivity to ACh was greater than that of young endothelium. The increased sensitivity in the present study appears to arise from the increased sensitivity of each cell in the population, as the fraction of cells activated at lower ACh concentrations was increased.

While the sensitivity of the cells to ACh increased, neither the percentage of cells that had oscillatory Ca^2+^ changes nor the frequency of the Ca^2+^ oscillations within cells differed between young and aged rats. This observation suggests that, when activated, cells responded in a similar way. However, in aged animals, the temporal spread of the Ca^2+^ responses was significantly larger than that occurring in young animals. The increased temporal spread of Ca^2+^ responses of young animals may reflect altered ACh diffusion through the arterial wall or differences in the mechanisms that initiate a Ca^2+^ rise in each cell.

### Imaging Approaches to Study Vascular Endothelium

To study pressure effects on the endothelium, we used a novel GRIN imaging system to visualize Ca^2+^ changes in large numbers of endothelial cells from inside arteries maintained in a physiological configuration. The GRIN imaging system differs substantially from other approaches used to measure Ca^2+^ changes in the endothelium in that it provides direct visualization of the endothelium from inside arteries that are in a physiological configuration. Several other approaches (photometric, widefield, and confocal microscopy) have previously been used to assess Ca^2+^ signaling in the endothelium. Each approach has advantages and disadvantages, and the choice of the technique requires careful consideration of the relative merits and limitations of each approach. For example, some investigations have utilized photometry to study endothelial Ca^2+^ signaling in isolated tubes of endothelium which lack any potential influence from smooth muscle cells [20,22,23,57]. Photometry may allow high-speed data collection and acquisition protocols in excess of 10 min [23]. However, photometric signals are integrated over the entire FOV and represent the averaged response of the entire population of cells. The subtleties and complexities of subcellular endothelial Ca^2+^ signals and how these contribute on a global scale [46,55,58], and, indeed, variation between responses of individual cells [20] or groups of cells [38], cannot be determined from photometric measures. This is particularly limiting when cells respond to a stimulus in a temporally dispersed manner, and when the extent of dispersion is related to stimulus levels [25,38].

In contrast to photometry, confocal imaging provides spatial information regarding the behavior of individual cells and an ability to selectively focus at some depth into tissue. Confocal microscopy has been used to reveal subcellular, localized endothelial Ca^2+^ signals in en face endothelial preparations [55,58] and also spontaneous Ca^2+^ signals in the endothelium of intact, pressurized arteries [46,59]. Most recently, intravital confocal imaging has been used to study spontaneous, localized Ca^2+^ signals in the mesentery of anesthetized mice [54]. However, in intact pressurized arteries, there is often a limited number of cells in focus because of the curvature of the artery and the small depth of the focal plane associated with high numerical aperture lenses (∼700 nm in the above paper with a 1.3 NA lens). Due to the small depth of field, small movements, which are hard to avoid in the intact pressurized arteries, can shift those cells being imaged outside the focal plane [54] and limit the data that can be gathered from any acquisition. The curvature of a pressurized artery further limits the effective FOV to a thin strip along the length of the artery which is typically a few cells wide. This could be partially overcome with high-speed focus scanning synchronized to the confocal scan at the expense of significant experimental complexity. However, such an approach has not been attempted to visualize the endothelium. Furthermore, whilst prolonged imaging is not possible with confocal imaging due to photobleaching of fluorescent indicators, widefield fluorescence microscopy using highly efficient electron multiplication CCD cameras and low-excitation light intensity can be utilized for prolonged (>5 min) and repeated endothelial imaging. This allows lengthy experimental protocols, for example full noncumulative concentration-response relationships to be gathered from a single field of endothelium [38,60].

The GRIN imaging approach used in the present study has a wide FOV (500 µm diameter) with a depth of focus (∼60 µm) that is large enough to maintain the focus on a curved, pressurized artery – allowing hundreds of cells to be visualized simultaneously. Because of the large depth of focus, GRIN imaging is also less prone to movement artifacts than confocal imaging. Thus, the present GRIN system is particularly useful for the study of large arteries from inside the lumen, and confocal imaging is useful for studies of small artery endothelium through the vascular wall. The lateral resolution of the GRIN system (4 µm), whilst more than adequate to study the subcellular progression of Ca^2+^ waves within individual cells, is lower than the than reported theoretical, diffraction-limited resolution of confocal microscopy (∼250 nm). However, in practice, the resolution achieved in confocal studies is often considerably lower than this theoretical value but, unfortunately, rarely quantified. In some cases, insufficient details are reported to allow an assessment of whether the optical systems used are capable of achieving diffraction-limited, as opposed to detector-limited, resolution.

When resolution has been quantified in confocal studies, this has most often been done using fluorescence beads placed directly on the coverslip. This method provides an artificially enhanced impression of resolution particularly in the following cases: (1) when high numerical aperture oil immersion lenses are used on inverted microscopes to image tissue through oil, a coverslip, and physiological saline; (2) when water immersion lenses are used on an inverted microscope to image through air, a coverslip, and physiological saline; and (3) when water immersion lenses are used on an upright microscope to image through saline and the vascular wall. For example, when oil immersion lenses are focused onto tissue through oil, a coverslip, and physiological saline, the refractive index mismatches, because the various materials cause severe expansion in the focal volume leading to loss of resolution [61]. This loss of resolution is compounded in the case where the confocal system must additionally focus through biological tissue. All of the small local changes in refractive indices (i.e., cell membranes and fat deposits) cause major changes in the light path, and result in aberration [62] and loss of resolution [63]. Such degradation in resolution is the main stimulus promoting the growth of the field of adaptive optics for both beam-scanned and widefield systems [64].

Thus, when imaging intact biological tissue one has to make compromises: photometry has the potential for high-speed, long-duration detection but without spatial information; traditional widefield microscopy enables long-duration imaging with high resolution but cannot focus through tissue; confocal or multiphoton, microscopy provides high spatial resolution which can be at depth but suffers from photobleaching and a low depth of field. Thus, none of these traditional microscopy techniques are well suited to imaging the endothelium of intact, curved vessels. GRIN microendoscopy thus fills the gap left by other imaging options – providing fast, wide FOV and subcellular resolution of the endothelium of intact vessels under a physiological mechanical load.

### Physiological Significance

Ca^2+^ signaling is a constituent endothelial signaling pathway and a critical regulator of endothelial function. For example, a rise in endothelial [Ca^2+^]_i_ is generally considered essential for the agonist-induced production of NO [16,17,18]. Endothelial dysfunction, which includes decreased NO production and vasodilation to ACh, has been demonstrated in several studies of aged animals [5,65,66] and aged humans [67]. Given the decreased NO production (a Ca^2+^-dependent process) and vasodilation to ACh, it is unexpected to measure an increased Ca^2+^ signal in the endothelium with increased age. The greater rise in [Ca^2+^]_i_ during muscarinic activation has been proposed to compensate for age-related reductions in NO bioavailability [68,69,70] by promoting eNOS activation [22].

Alterations in the mechanical properties of blood vessels and altered production of endothelium-derived vasoactive factors such as endothelin [71], prostaglandin H_2_, thromboxane A_2_[72], and NO [5,6,7,8] are each proposed to mediate the age-related decrease in endothelium-dependent vasodilation. Our results show decreased pressure sensitivity with age. Thus, suppression of endothelial Ca^2+^ signals by increased pressure in young animals is attenuated with age. As a result of the decreased pressure sensitivity, Ca^2+^ signaling would be maintained as pressure is increased and may help minimize the physiological consequences of a decrease in eNOS activity with age [5,7] Thus, while aging is the summation of many, mostly detrimental changes to the function of the cardiovascular system, agonist-evoked endothelial Ca^2+^ signaling is paradoxically increased and may serve to offset other decreases in endothelial function, such as decreased eNOS activity, and maintain vascular contractile function.

## Disclosure Statement

The authors have no conflicts of interest to disclose.

## References

[1] Matz RL, Alvarez de Sotomayor M, Schott C, Andriantsitohaina R (2003). Preservation of vascular contraction during ageing: dual effect on calcium handling and sensitization. Br J Pharmacol.

[2] Shipley RD, Muller-Delp JM (2005). Aging decreases vasoconstrictor responses of coronary resistance arterioles through endothelium-dependent mechanisms. Cardiovasc Res.

[3] Ishihata A, Ogaki T, Aita T, Katano Y (2005). Role of prostaglandins in urotensin II-induced vasodilatation in the coronary arteries of aged rats. Eur J Pharmacol.

[4] Ibarra M, López-Guerrero JJ, Mejía-Zepeda R, Villalobos-Molina R (2006). Endothelium-dependent inhibition of the contractile response is decreased in aorta from aged and spontaneously hypertensive rats. Arch Med Res.

[5] Chou T-C, Yen M-H, Li C-Y, Ding Y-A (1998). Alterations of nitric oxide synthase expression with aging and hypertension in rats. Hypertension.

[6] Perrier E, Fournet-Bourguignon M-P, Royere E, Molez S, Reure H, Lesage L (2009). Effect of uncoupling endothelial nitric oxide synthase on calcium homeostasis in aged porcine endothelial cells. Cardiovasc Res.

[7] Yang Y-M, Huang A, Kaley G, Sun D (2009). eNOS uncoupling and endothelial dysfunction in aged vessels. Am J Physiol Heart Circ Physiol.

[8] Puca AA, Carrizzo A, Ferrario A, Villa F, Vecchione C (2012). Endothelial nitric oxide synthase, vascular integrity and human exceptional longevity. Immun Ageing.

[9] Virmani R, Avolio AP, Mergner WJ, Robinowitz M, Herderick EE, Cornhill JF (1991). Effect of aging on aortic morphology in populations with high and low prevalence of hypertension and atherosclerosis. Comparison between occidental and Chinese communities. Am J Pathol.

[10] Nichols WW, O'Rourke MF, Vlachopoulos C (2011). McDonald's Blood Flow in Arteries: Theoretical, Experimental and Clinical Principles.

[11] Kalaria RN (1996). Cerebral vessels in ageing and Alzheimer's disease. Pharmacol Ther.

[12] Bramwell JC, Hill AV (1922). The velocity of the pulse wave in man. Proc R Soc Lond B.

[13] Vlachopoulos C, Aznaouridis K, Stefanadis C (2010). Prediction of cardiovascular events and all-cause mortality with arterial stiffness: a systematic review and meta-analysis. J Am Coll Cardiol.

[14] Safar ME, Henry O, Meaume S (2002). Aortic pulse wave velocity: an independent marker of cardiovascular risk. Am J Geriatr Cardiol.

[15] Reference Values for Arterial Stiffness' Collaboration (2010). Determinants of pulse wave velocity in healthy people and in the presence of cardiovascular risk factors: “establishing normal and reference values. ” Eur Heart J.

[16] Kuchan MJ, Frangos JA (1994). Role of calcium and calmodulin in flow-induced nitric oxide production in endothelial cells. Am J Physiol.

[17] Schmidt HHHW, Pollock JS, Nakane M, Förstermann U, Murad F (1992). Ca^2+^/calmodulin-regulated nitric oxide synthases. Cell Calcium.

[18] Falcone JC, Kuo L, Meininger GA (1993). Endothelial cell calcium increases during flow-induced dilation in isolated arterioles. Am J Physiol Heart Circ Physiol.

[19] Tran QK, Watanabe H (2006). Calcium signalling in the endothelium.. Handb Exp Pharmacol.

[20] Socha MJ, Domeier TL, Behringer EJ, Segal SS (2012). Coordination of intercellular Ca^2+^ signaling in endothelial cell tubes of mouse resistance arteries. Microcirculation.

[21] Prendergast C, Quayle J, Burdyga T, Wray S (2014). Atherosclerosis differentially affects calcium signalling in endothelial cells from aortic arch and thoracic aorta in apolipoprotein E knockout mice. Physiol Rep.

[22] Socha MJ, Boerman EM, Behringer EJ, Shaw RL, Domeier TL, Segal SS (2015). Advanced age protects microvascular endothelium from aberrant Ca^2+^ influx and cell death induced by hydrogen peroxide. J Physiol.

[23] Behringer EJ, Segal SS (2015). Membrane potential governs calcium influx into microvascular endothelium: integral role for muscarinic receptor activation. J Physiol.

[24] Zhang DX, Mendoza SA, Bubolz AH, Mizuno A, Ge Z-D, Li R (2009). Transient receptor potential vanilloid type 4-deficient mice exhibit impaired endothelium-dependent relaxation induced by acetylcholine in vitro and in vivo. Hypertension.

[25] Wilson C, Saunter CD, Girkin JM, McCarron JG (2015). Pressure-dependent regulation of Ca^2+^ signalling in the vascular endothelium. J Physiol.

[26] Hishikawa K, Nakaki T, Suzuki H, Saruta T, Kato R (1992). Transmural pressure inhibits nitric oxide release from human endothelial cells. Eur J Pharmacol.

[27] Huang A, Sun D, Kaley G, Koller A (1998). Superoxide released to high intra-arteriolar pressure reduces nitric oxide-mediated shear stress- and agonist-induced dilations. Circ Res.

[28] Zhao Y, Flavahan S, Leung SWS, Xu A, Vanhoutte PM, Flavahan NA (2015). Elevated pressure causes endothelial dysfunction in mouse carotid arteries by increasing local angiotensin signaling. Am J Physiol Heart Circ Physiol.

[29] Jurva JW, Phillips SA, Syed AQ, Syed AY, Pitt S, Weaver A (2006). The effect of exertional hypertension evoked by weight lifting on vascular endothelial function. J Am Coll Cardiol.

[30] Phillips SA, Das E, Wang J, Pritchard K, Gutterman DD (2011). Resistance and aerobic exercise protects against acute endothelial impairment induced by a single exposure to hypertension during exertion. J Appl Physiol.

[31] Altschul R (1954). Endothelium. Its Development, Morphology, Function, and Pathology..

[32] Alba C, Vidal L, Díaz F, Villena A, de Vargas IP (2004). Ultrastructural and quantitative age-related changes in capillaries of the dorsal lateral geniculate nucleus. Brain Res Bull.

[33] Deanfield JE, Halcox JP, Rabelink TJ (2007). Endothelial function and dysfunction testing and clinical relevance. Circulation.

[34] Mchedlishvili G (1986). Arterial behavior and blood circulation in the brain..

[35] Faraci FM, Heistad DD (1990). Regulation of large cerebral arteries and cerebral microvascular pressure. Circ Res.

[36] Sengupta P (2013). The laboratory rat: relating its age with human's. Int J Prev Med.

[37] Craig J, Martin W (2012). Dominance of flow-mediated constriction over flow-mediated dilatation in the rat carotid artery. Br J Pharmacol.

[38] Wilson C, Saunter CD, Girkin JM, McCarron JG (2016). Clusters of specialized detector cells provide sensitive and high fidelity receptor signalling in the intact endothelium. FASEB J.

[39] Edelstein A, Amodaj N, Hoover K, Vale R, Stuurman N (2010). Computer control of microscopes using µManager.. Curr Protoc Mol Biol.

[40] Popp R, Fleming I, Busse R (1998). Pulsatile stretch in coronary arteries elicits release of endothelium-derived hyperpolarizing factor: a modulator of arterial compliance. Circ Res.

[41] Muller JM, Davis MJ, Kuo L, Chilian WM (1999). Changes in coronary endothelial cell Ca^2+^ concentration during shear stress- and agonist-induced vasodilation. Am J Physiol.

[42] Marchenko SM, Sage SO (2000). Effects of shear stress on [Ca^2+^]_i_ and membrane potential of vascular endothelium of intact rat blood vessels. Exp Physiol.

[43] Sun D, Huang A, Recchia FA, Cui Y, Messina EJ, Koller A (2001). Nitric oxide-mediated arteriolar dilation after endothelial deformation. Am J Physiol Heart Circ Physiol.

[44] Duza T, Sarelius IH (2004). Localized transient increases in endothelial cell Ca^2+^ in arterioles in situ: implications for coordination of vascular function. Am J Physiol Heart Circ Physiol.

[45] Gündüz F, Meiselman HJ, Başkurt OK (2008). High intravascular pressure attenuates vascular dilation responses of small mesenteric arteries in the rat. Circ J.

[46] Bagher P, Beleznai T, Kansui Y, Mitchell R, Garland CJ, Dora KA (2012). Low intravascular pressure activates endothelial cell TRPV4 channels, local Ca^2+^ events, and IK_Ca_ channels, reducing arteriolar tone. Proc Natl Acad Sci USA.

[47] Blackwell KA, Sorenson JP, Richardson DM, Smith LA, Suda O, Nath K (2004). Mechanisms of aging-induced impairment of endothelium-dependent relaxation: role of tetrahydrobiopterin. Am J Physiol Heart Circ Physiol.

[48] Takenouchi Y, Kobayashi T, Matsumoto T, Kamata K (2009). Gender differences in age-related endothelial function in the murine aorta. Atherosclerosis.

[49] Wang YX, Halks-Miller M, Vergona R, Sullivan ME, Fitch R, Mallari C (2000). Increased aortic stiffness assessed by pulse wave velocity in apolipoprotein E-deficient mice. Am J Physiol Heart Circ Physiol.

[50] Hashimoto M, Gamoh S, Hossain S, Okunishi H, Shimoura K, Yasui Y (1998). Age-related changes in aortic sensitivity to noradrenaline and acetylcholine in rats. Clin Exp Pharmacol Physiol.

[51] Kano Y, Tanabe T, Nagasawa J, Mizuta T (2000). Effect of age on rat aortic responses to acetylcholine and nitric oxide donor (NOC-18). Res Commun Mol Pathol Pharmacol.

[52] Crespo MJ, Escobales N, Rodríguez-Sargent C (1996). Endothelial dysfunction in the San Juan hypertensive rat: possible role of the nitric oxide synthase. J Cardiovasc Pharmacol.

[53] Chinellato A, Pandolfo L, Ragazzi E, Zambonin MR, Froldi G, De Biasi M (1991). Effect of age on rabbit aortic responses to relaxant endothelium-dependent and endothelium-independent agents. Blood Vessels.

[54] Boerman EM, Everhart JE, Segal SS (2016). Advanced age decreases local calcium signaling in endothelium of mouse mesenteric arteries in vivo. Am J Physiol Heart Circ Physiol.

[55] Ledoux J, Taylor MS, Bonev AD, Hannah RM, Solodushko V, Shui B (2008). Functional architecture of inositol 1,4,5-trisphosphate signaling in restricted spaces of myoendothelial projections. Proc Natl Acad Sci USA.

[56] Busse R, Mülsch A (1990). Calcium-dependent nitric oxide synthesis in endothelial cytosol is mediated by calmodulin. FEBS Lett.

[57] Socha MJ, Hakim CH, Jackson WF, Segal SS (2011). Temperature effects on morphological integrity and Ca^2+^ signaling in freshly isolated murine feed artery endothelial cell tubes. Am J Physiol Heart Circ Physiol.

[58] Sonkusare SK, Bonev AD, Ledoux J, Liedtke W, Kotlikoff MI, Heppner TJ (2012). Elementary Ca^2+^ signals through endothelial TRPV4 channels regulate vascular function. Science.

[59] Kansui Y, Garland CJ, Dora KA (2008). Enhanced spontaneous Ca^2+^ events in endothelial cells reflect signalling through myoendothelial gap junctions in pressurized mesenteric arteries. Cell Calcium.

[60] Wilson C, Lee M, McCarron JG (2016). Acetylcholine released by endothelial cells facilitates flow-mediated dilatation.. J Physiol.

[61] Booth MJ, Neil MAA, Wilson T (1998). Aberration correction for confocal imaging in refractive-index-mismatched media. J Microsc.

[62] Schwertner M, Booth MJ, Wilson T (2004). Characterizing specimen induced aberrations for high NA adaptive optical microscopy. Opt Express.

[63] Chaigneau E, Wright AJ, Poland SP, Girkin JM, Silver RA (2011). Impact of wavefront distortion and scattering on 2-photon microscopy in mammalian brain tissue. Opt Express.

[64] Girkin JM, Poland S, Wright AJ (2009). Adaptive optics for deeper imaging of biological samples. Curr Opin Biotechnol.

[65] Konishi M, Su C (1983). Role of endothelium in dilator responses of spontaneously hypertensive rat arteries. Hypertension.

[66] Lüscher TF, Vanhoutte PM (1986). Endothelium-dependent contractions to acetylcholine in the aorta of the spontaneously hypertensive rat. Hypertension.

[67] Taddei S, Virdis A, Mattei P, Ghiadoni L, Gennari A, Fasolo CB (1995). Aging and endothelial function in normotensive subjects and patients with essential hypertension. Circulation.

[68] Taddei S, Virdis A, Ghiadoni L, Salvetti G, Bernini G, Magagna A (2001). Age-related reduction of NO availability and oxidative stress in humans. Hypertension.

[69] Seals DR, Jablonski KL, Donato AJ (2011). Aging and vascular endothelial function in humans. Clin Sci (Lond).

[70] Muller-Delp JM, Gurovich AN, Christou DD, Leeuwenburgh C (2012). Redox balance in the aging microcirculation: new friends, new foes, and new clinical directions. Microcirculation.

[71] Barton M (2014). Aging and endothelin: determinants of disease. Life Sci.

[72] Tang EHC, Vanhoutte PM (2008). Gene expression changes of prostanoid synthases in endothelial cells and prostanoid receptors in vascular smooth muscle cells caused by aging and hypertension. Physiol Genomics.

